# Increased DNA Polymerase Epsilon Catalytic Subunit Expression Predicts Tumor Progression and Modulates Tumor Microenvironment of Hepatocellular Carcinoma

**DOI:** 10.7150/jca.64765

**Published:** 2022-06-03

**Authors:** Haijia Tang, Xiaoxin Hu, Fujiang Xu, Kefei Lin, Wenhao Xu, Haineng Huang, Hailiang Zhang, Yu Xiao, Dongdong Sun, Wangrui Liu, Shiyin Wei

**Affiliations:** 1 Department of Integrated Medicine, Nanjing University of Chinese Medicine, Nanjing, 210000, China; 2 Department of Radiology, Fudan University Shanghai Cancer Center, Shanghai, China; 3 Department of Urology, Fudan University Shanghai Cancer Center, School of Life Sciences, Fudan University, Shanghai, China; 4 Department of Urology, PKUCare CNOOC Hospital,Tianjin,300452, China; 5 Affiliated Hospital of Youjiang Medical University for Nationalities, Guangxi, China; 6 School of Integrated Chinese and Western Medicine, Nanjing University of Chinese Medicine, Nanjing 210023, China; 7 Department of Interventional Oncology, Renji Hospital, Shanghai Jiao Tong University School of Medicine, Shanghai, China

**Keywords:** POLE, liver hepatocellular carcinoma, tumor immune microenvironment, prognosis

## Abstract

**Backgrounds:** Liver hepatocellular carcinoma (HCC) is one of the most common cancers worldwide, and POLE, playing an important role in maintaining genetic stability, is closely connected with cancer prognosis. This study aimed to explore the significance role of POLE in HCC prognosis, clinical treatment and tumor immune microenvironment based on large-scale multiply cohorts.

**Methods:** First, we found that the expression of POLE was prominently higher in tumor tissues than in normal tissues, and was closely related to clinical stage, grade and patient outcomes. Second, we found that patients with high POLE expression had significantly aggressive progression, indicating effective predictive role of POLE expression for Asian, male, low-risk HCC patients. Additionally, POLE mutation frequency was detected in several datasets with available genomic-wide data.

**Results:** 130 HCC samples from real-world Renji cohort were included to demonstrate that elevated POLE expression was significantly connected to the invasive progression and poor prognosis. More importantly, the expression of POLE was closely related to the anti-tumoral activity of immune cells and immune checkpoints expression, suggesting a bright prospect of POLE as a predictive biomarker in immunotherapy.

**Conclusion:** In conclusion, this study revealed that high expression of POLE significantly correlated to the malignant progression, poor prognosis and anti-tumoral activity of immune cells in HCC. Thus, POLE could function as a biomarker for the early diagnosis, prognosis, immune-excluded tumor microenvironment and response to immunotherapy of HCC.

## Introduction

Liver cancer is the fifth most common cancer worldwide, and liver hepatocellular carcinoma (HCC) is the main manifestation of primary liver cancer with low survival rate, high recurrence rate and poor prognosis 1, 2. Accounting for 70% to 90% of all pathological types, HCC is one of the most common malignant tumors in humans, ranking third in the cause of cancer-related death 3, and the majority of patients are diagnosed at an advanced stage 4.

Traditional treatment methods include surgical resection, liver transplantation, vascular intervention, radiofrequency ablation and drug combination therapy 5. However, a significant number of patients with (HCC) suffered from drug resistance 6, and immune escape of HCC was still a difficult problem in tumor therapy 7. So there has been research focus on the immune microenvironment of cancers 8. Previously, various immunotherapy methods have been developed, which mainly rely on immune cells within or outside of the tumor microenvironment (TME) to specifically target and attack cancer cells 9. Also, immune checkpoint inhibitor therapy has been employed, and hub genes like POLE, DK1, CDC20 and CCNB1 have been found as biomarkers of progression in HCC patients 10-12. However, the immune microenvironment of HCC has not been fully explored 13. We still need to find biomarkers with diagnostic and prognostic value to construct a more comprehensive view of therapeutic methods based on the immune microenvironment of HCC 14-16.

DNA damage repair (DDR) pathway consists of multiple interconnected cellular signaling networks which are activated upon DNA damage 17. DDR is associated with genomic instability, tumor mutational burden and immune cell function in HCC 18. DNA Polymerase Epsilon (POLE), a Protein Coding gene, is involved in DNA repair and chromosomal DNA replication, playing an important role in maintaining genetic stability in the process of DNA replication 19. In addition, POLE involved in the mechanism by which the DNA damage repair (DDR) pathway coordinates DNA repair and apoptosis, thereby regulating the response of cancer patients receiving radiotherapy, chemotherapy, and targeted therapy, such as clear cell renal cell carcinoma 20. Therefore, POLE is expected to become a target for new anti-tumor therapeutic strategies and prognostic markers 21.

## Methods

### Patients and tissue samples from online databases and real-world cohorts

The large-scale cohorts in this study included RNA sequencing data, clinical and pathological data for 421 HCC patients collected from The Cancer Genome Atlas (TCGA, http://www.cancer.gov) and 442 patients with HCC from International Cancer Genome Consortium (ICGC, https://dcc.icgc.org/) database. HCC and normal samples of 130 patients with HCC was obtained during operation from Renji cohort.

The Renji hospital consisted of 130 patients diagnosed with HCC, from 2010 to 2020. Clinicopathological data were collected from pathology reports or electronic medical records. Samples of HCC and normal liver tissues were collected during surgery and then processed and stored at the tissue bank before experiments.

### Immunohistochemistry (IHC) staining analysis

IHC was performed with an anti-POLE antibody-N-terminal (ab226848) at a 1:1000 dilution. IHC staining was conducted in accordance with the manufacturer's instructions as previously described 22. According to the IHC staining intensity and density, multiply the two scores to obtain the overall IHC score (from 0 to 12). Score 0 to 3 indicates negative staining and score 4 to 12 indicates positive staining of each tissue.

### Abundance and frequency of POLE mutations in HCC

To investigate the potential role of POLE, we analyzed the mutation abundance and frequency of POLE expression in HCC using cBioportal for cancer genomics (http://www.cbioportal.org/). Significantly elevated genes between POLE mutated and unmutated genes were screened and identified by the Limma R package. The frequency of typical gene mutations in HCC according to differential POLE expression was also analyzed.

### Analysis of immune infiltration in the tumor microenvironment

CIBERSORT uses a deconvolution algorithm to estimate the composition and abundance of immune cells in mixed cells based on transcriptome data (https://cibersort.stanford.edu). By using the CIBERSORT algorithm, we obtained a variety of immune infiltrating lymphocytes with different infiltration degrees. We divided HCC patients into POLE^high^ group and POLE^low^ group according to the median of POLE expression. Differential infiltrated lymphocytes were assessed using Wilcoxon rank sum test.

### Statistics analysis

The relationship between pathological factors and patient survival was discussed by Sankey diagram. In order to determine the statistical significance of differential POLE expression between tumor tissues and normal tissues, Kaplan Meier curves were used to assess the significance of progression-free survival (PFS) and overall survival (OS) benefits in different POLE expression groups and all subgroups by the Kaplan-Meier Plotter (http://kmplot.com/analysis/index). We performed univariate and multivariate Cox regression analyses, and built the nomogram to predict the X-year overall recurrence and to calculate the risk of recurrence for individual patients. Then, functional and pathway enrichment analyses of KEGG pathways and GO pathways were performed and represented in bubble charts.

## Results

### Expression of POLE correlated with aggressive clinicopathological parameters for HCC patients

**Figure 1:**
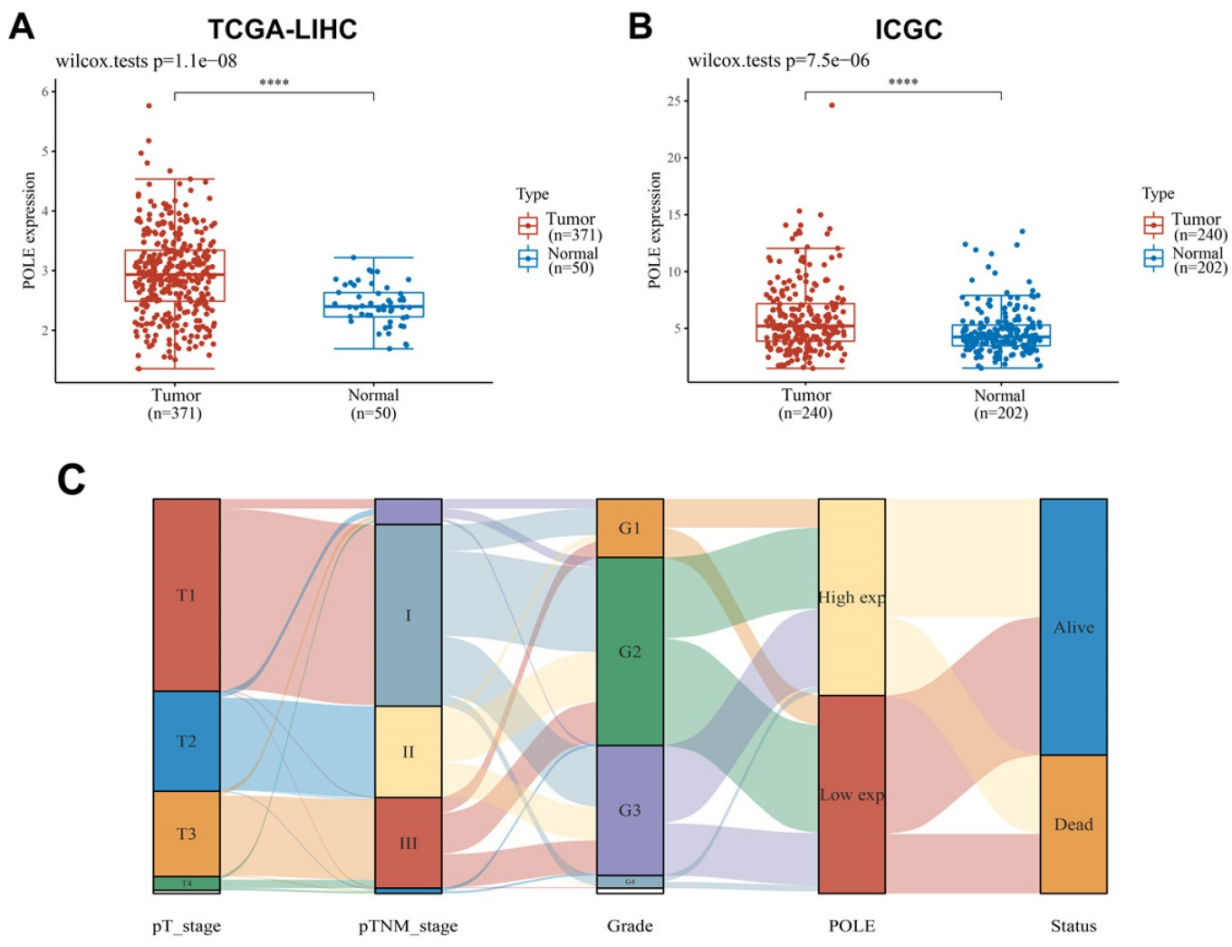
** Expression of POLE correlated with aggressive clinicopathological parameters for HCC patients. (A)** We selected samples from HCC patients from TCGA and ICGC databases and compared POLE expression in normal tissue and tumor tissue. **(B)** Sankey diagram was employed to demonstrate relationship between tumor grades, stages, POLE expression level and survival.

With the samples of liver hepatocellular carcinoma (HCC) in TCGA database (n=421) and ICGC database (n=442), we compared the expression level of POLE in normal tissues and tumor tissues, and found that POLE expression was significantly higher in tumor tissues **(Figure 1A, B)**. The correlation between tumor stages, grades, POLE expression levels and survival status were demonstrated in Sankey diagram. Early-stage and low tumor grade and HCC patients tended are related to low expression of POLE, and advanced stages and high tumor grades are associated with increased POLE expression **(Figure 1C)**.

### Prognostic role of POLE in HCC of different subgroups

**Figure 2:**
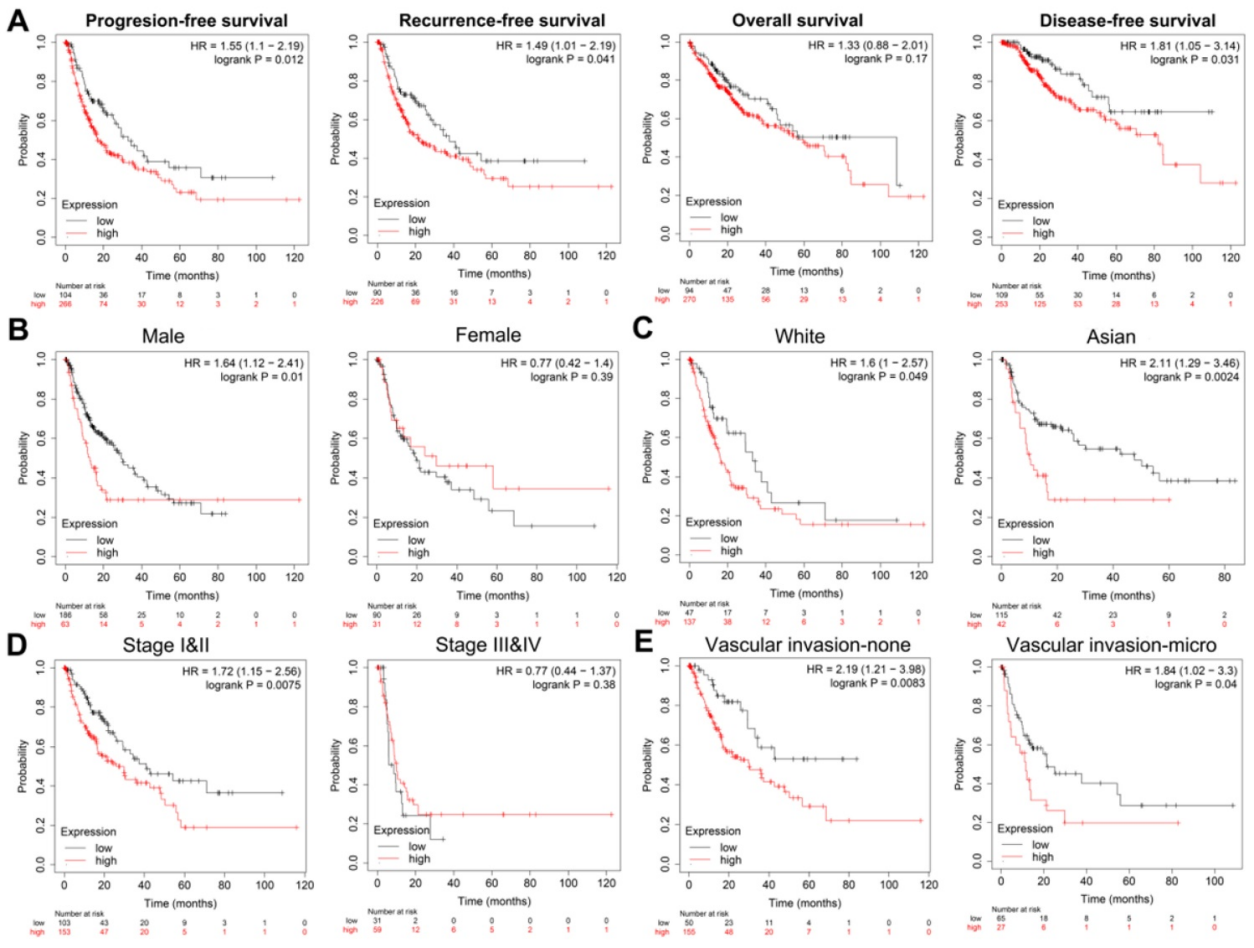
**Prognostic role of POLE in HCC of different subgroups.** PFS, RFS, OS and DFS analysis of HCC patients were performed, and PFS in subgroups of gender, race, clinical stage and vascular invasion were also represented by KM curve using Kaplan-Meier Plotter.

Next, we performed Kaplan-Meier analysis of HCC patients from TCGA cohort (n=370). First, in Progression-free survival (PFS), recurrence-free survival (RFS), overall survival (OS) and Disease-free survival (DFS) analyses, we found that the survival rate of patients with high POLE expression was lower than that of patients with low POLE expression **(Figure 2A)**. Then we conducted subgroup survival analyses of POLE expression to compare the effects of gender, race, clinical stage and vascular invasion on PFS **(Figure 2B-E)**. The pattern of worse PFS with high POLE expression was significant in subgroups of male, Asian, clinical stage 1 and 2 and no vascular infiltration group, suggesting possible interference of hormone level on the expression of POLE, and significance of POLE in early diagnosis of hepatocellular carcinoma.

### POLE significantly predicts PFS for HCC patients

**Figure 3:**
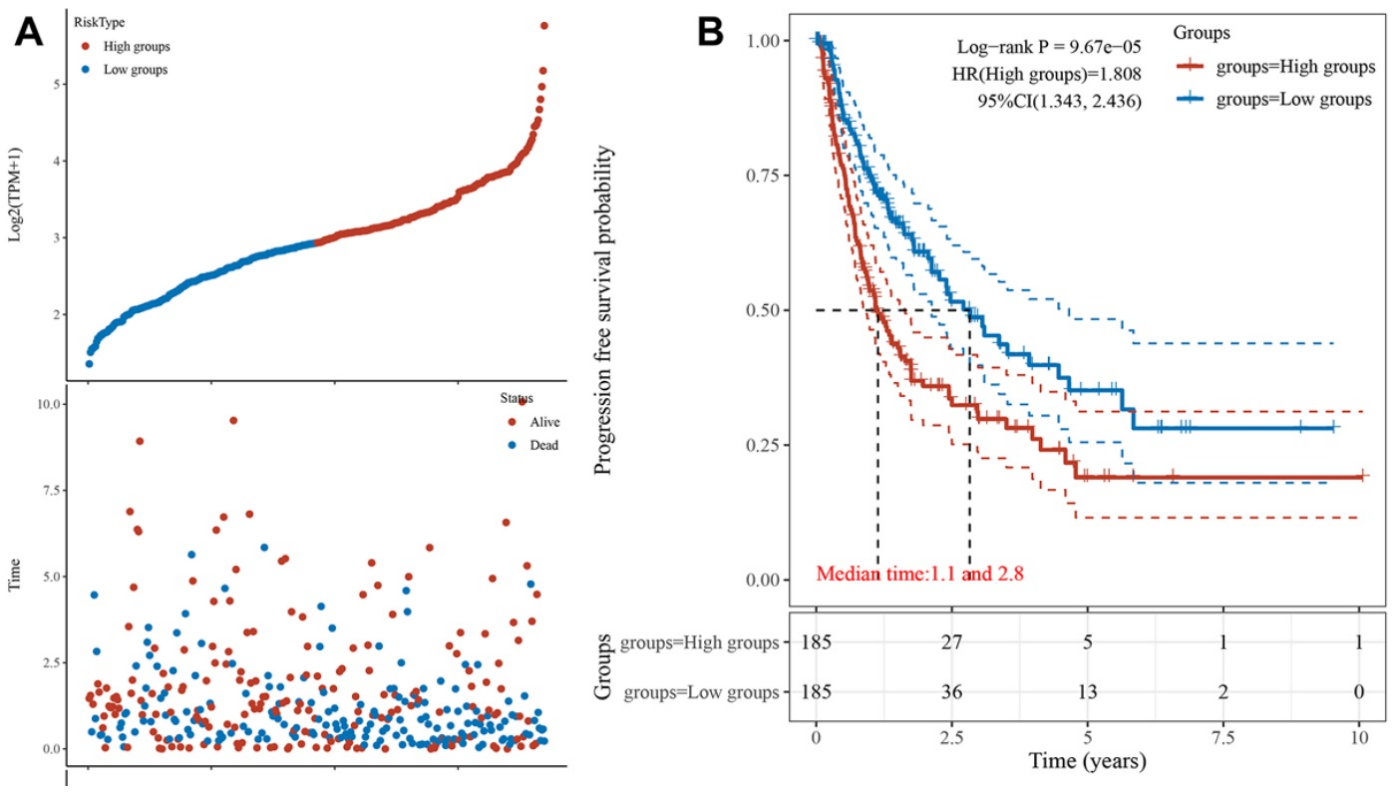
** POLE significantly predicts PFS for HCC patients.** We obtained the survival time of each sample and utilized the Kaplan-Meier curve with median survival to further explore correlation between the expression of POLE and survival of HCC patients.

Next, we obtained the PFS time of each sample and compared the high expression group with the low expression group, the result showed that HCC patients with higher POLE expression experienced a significantly increased risk of death **(Figure 3A)**. To further explore correlation between the expression of POLE and survival of HCC patients, the Kaplan-Meier curve with median survival of 1.1 years in the POLE^high^ group and 2.8 years in the POLE^low^ group also demonstrated worse PFS of patients with high POLE expression **(Figure 3B)**.

### Construction of nomogram and Cox regression analysis

**Figure 4:**
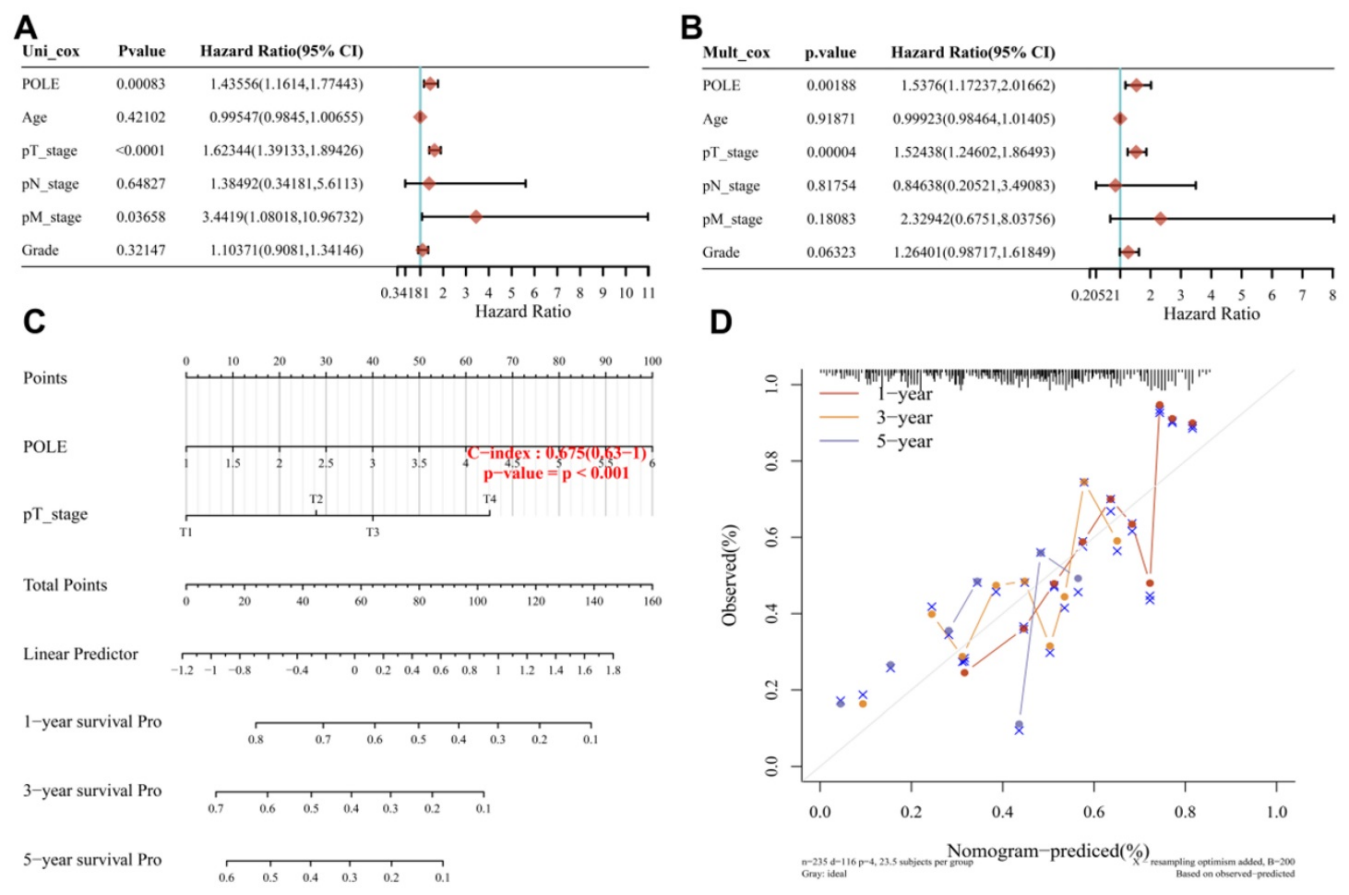
** Construction of nomogram and Cox regression analysis. (A)** We performed uni-Cox regression analysis to find factors associated with survival in HCC patients. **(B)** Mult_Cox regression was analysis was used to supplement the important factors influencing the survival of patients with hepatocellular carcinoma after the interaction of different factors. **(C)** A Nomogram with prediction model of POLE expression and pT stage was used to evaluated the prognosis of HCC patients (C-index=0.675, *p*<0.001). **(D)** The risk of recurrence of individual patients was calculated by Nomogram, using a graphical representation of factors we have found. The short-term prediction was more accurate.

**Figure 5:**
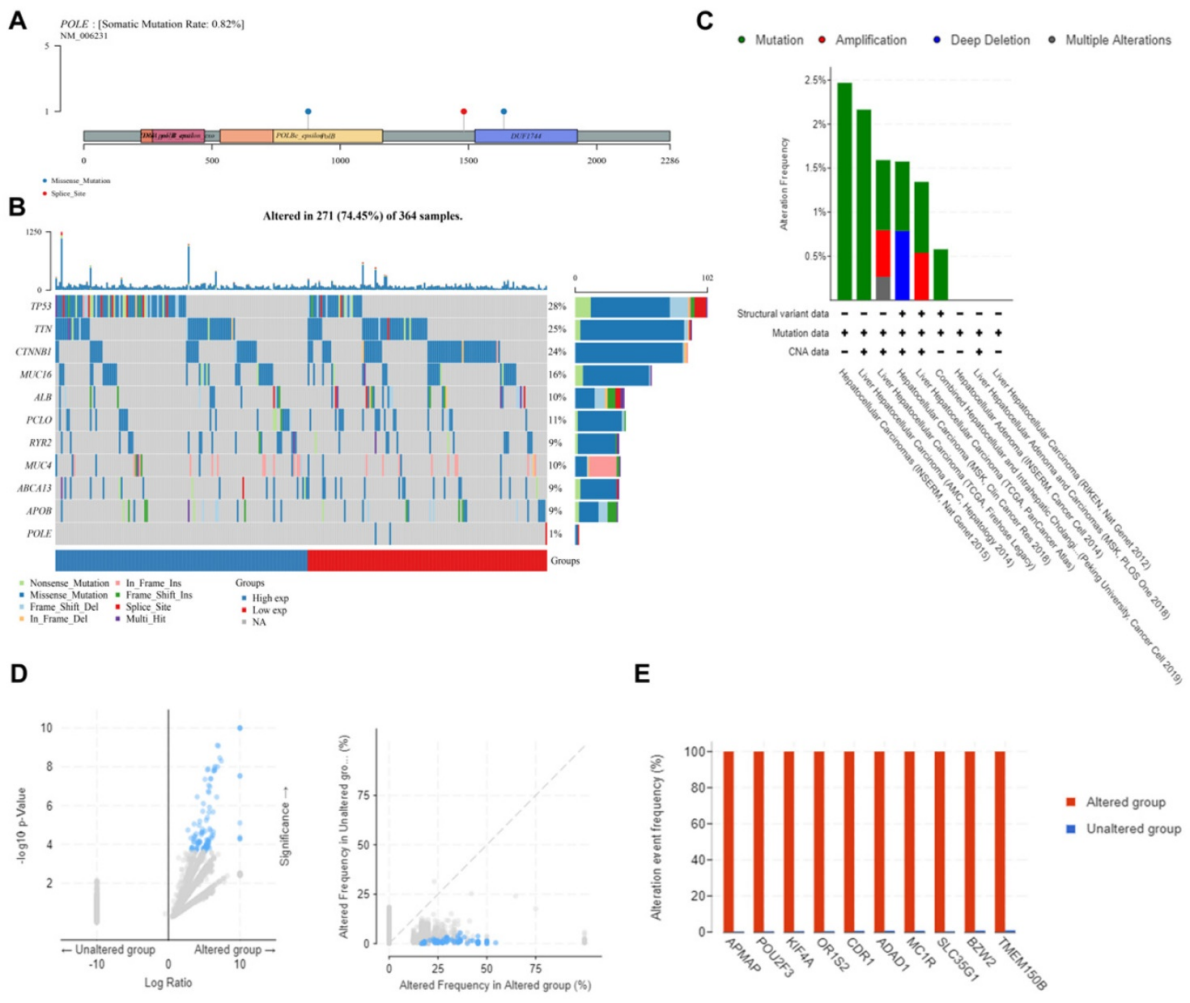
** Landscape of POLE mutation and altered genes in HCC. (A)** POLE mutation was found mainly because of the missense on POLBc_epsilonPolB and DUF1744. **(B)** Mutation frequency of common gene in HCC was explored according to differential POLE expression in 271 cases. **(C)** We exploited cBioportal investigate the frequency and pattern of POLE mutation in HCC in different cohorts. **(D-E)** Significantly elevated genes between POLE altered and unaltered group were screened and identified, including APMAP, POU2F3, KIF4A, OR1S2, CDR1, ADAD1, MC1R, SLC35G1, BZW2 and TMEM150B.

The univariate Cox regression analysis showed the close correlation between POLE and pT stage and the survival of HCC patients (*p*<0.001). And with interaction between these factors, POLE expression (HR=1.53, *p*=0.00188) and pT stage (HR=1.52, *p*<0.01) showed a significant effect on the prognosis of HCC in multivariate Cox regression **(Figure 4A, B)**. The graphical representation of the factors provided by nomogram was used to calculate the recurrence risk of individual patient according to the points related to each risk factor, and the model was more accurate in the prediction of short-term survival (C-index=0.675, *p*<0.001; **Figure 5C**). Then POLE expression and pT stage are included in a prediction model nomogram to evaluate the prognosis of HCC (*p*<0.001) **(Figure 4D)**. So far, our findings suggest a consistent relationship between high POLE expression and advanced clinicopathological stage.

### Landscape of POLE mutation and altered genes in HCC

Furthermore, we analyzed multi-omics data to explore the potential value of POLE mutation and other related genes. First, we found the mutation frequency was only 0.82% in samples from TCGA database. And the mutation was mainly because of the missense on POLBc_epsilonPolB and DUF1744 **(Figure 5A)**. Second, we analyzed the mutation frequency of common genes in HCC based on different POLE expression in 271 cases, including TP53, TTN, CTNNB1, MUC16, ALB, PCLO, RYR2, MUC4, ABCA13, APOB and POLE, with mutation frequency of TP53 ranking the first (28%). And POLE^high^ group has higher TP53 mutation rate than POLE^low^ group, suggesting that TP53 expression level plays an important role in clinical treatment and tumor prognosis **(Figure 5B)**. Then we obtained samples from cBioPortal to test the mutation frequency and types. In INSERM corhort, mutation rate could reach about 2.5%, frequency of mutation and copy number variation in AMC cohort reached 2.2%, mutation in TCGA cohort was around 1.6%, with amplification accounting for 0.5% and multiple alterations accounting for 0.25%, and in MSK cohort, mutation frequency reached 1.5%, with copy number deletion accounting for a half **(Figure 5C)**. The difference in gene expression between POLE altered and unaltered genes was still significant **(Figure 5D)**. So we investigated significant differences between the POLE altered and unaltered groups. Alteration frequency of common genes in altered group was significantly up-regulated, including APMAP, POU2F3, KIF4A, OR1S2, CDR1, ADAD1, MC1R, SLC35G1, BZW2 and TMEM150B **(Figure 5E)**.

### Differential expression genes and functional enrichment analysis

**Figure 6:**
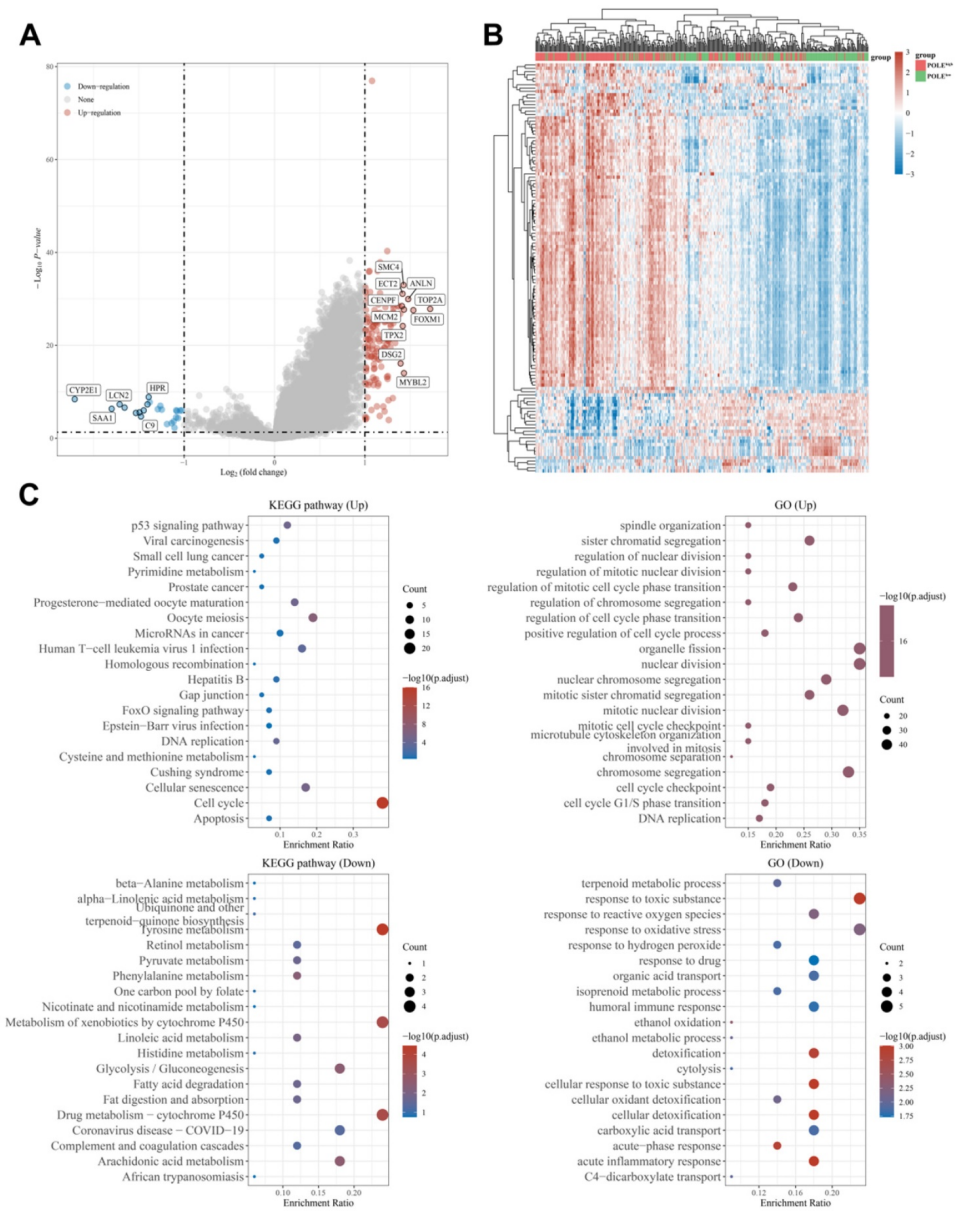
** Differential expression genes and functional enrichment analysis. (A-B)** Up-regulated and down-regulated genes in POLE high and low expression groups were identified. **(C)** Functional and pathway enrichment analyses and to obtain a bubble chart. Up-regulated and down-regulated pathways in KEGG database and GO database are listed in the chart.

In addition, we found out the up-regulated genes like SMC4, TOP2A, ECT2 and CENPF, and down-regulated genes like CYP2E1, SAA1 and LCN2 **(Figure 6A)**. Through the heat map, we can see the genes were mainly up-regulated in the POLE high-expression group, while the down-regulated genes accounted for the majority in the POLE low-expression group** (Figure 6B)**. Then, functional and pathway enrichment analyses of KEGG pathways and GO pathways were performed and represented in bubble charts. The result shows that change of up-regulated pathway in KEGG database was significantly enriched in cell cycle, changes of down-regulated pathway in KEGG database were mainly enriched in Tyrosine metabolism, metabolism of xenobiotics by cytochrome P450 and drug metabolism-cytochrome P450. In GO database, changes of up-regulated pathway were mostly enriched in organelle fission and nuclear division, changes of down-regulated pathway were existed in response to toxic substance and response to oxidative stress **(Figure 6C)**.

### Validation of differential POLE expression and its prognostic value in the Renji cohort

**Figure 7:**
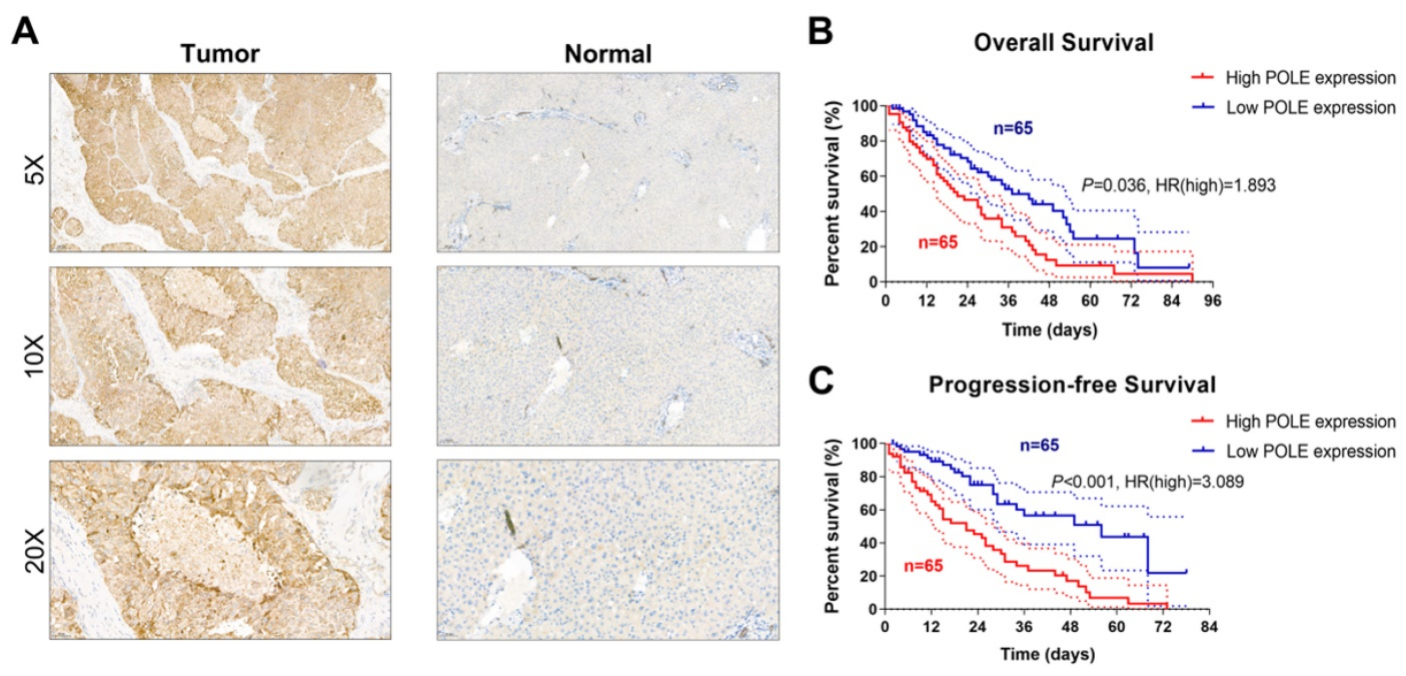
** Validation of differential POLE expression and its prognostic value in the Renji cohort. (A)** IHC analysis of samples from Renji cohort showed that expression of POLE was significantly higher in tumor tissues than in normal liver tissues. **(B-C)** Prognostic implications of POLE expression of 130 samples from Renji cohort.

To determine whether POLE expression is altered in HCC samples compared with normal liver tissue, we performed IHC staining analysis of tissues from the 130-patient Renji cohort, and increased POLE was found in tumor tissues** (Figure 7A)**. We found that expression of POLE was lower in normal tissues than in tumor tissues. Survival analysis of real-world cohort samples suggested that higher expression of POLE was significantly linked to worse OS (*p*=0.036, HR=1.893) and PFS (*p*<0.001, HR=3.089) for patients with HCC **(Figure 7B, C)**.

### Immunological response within HCC in accordance with POLE expression

**Figure 8:**
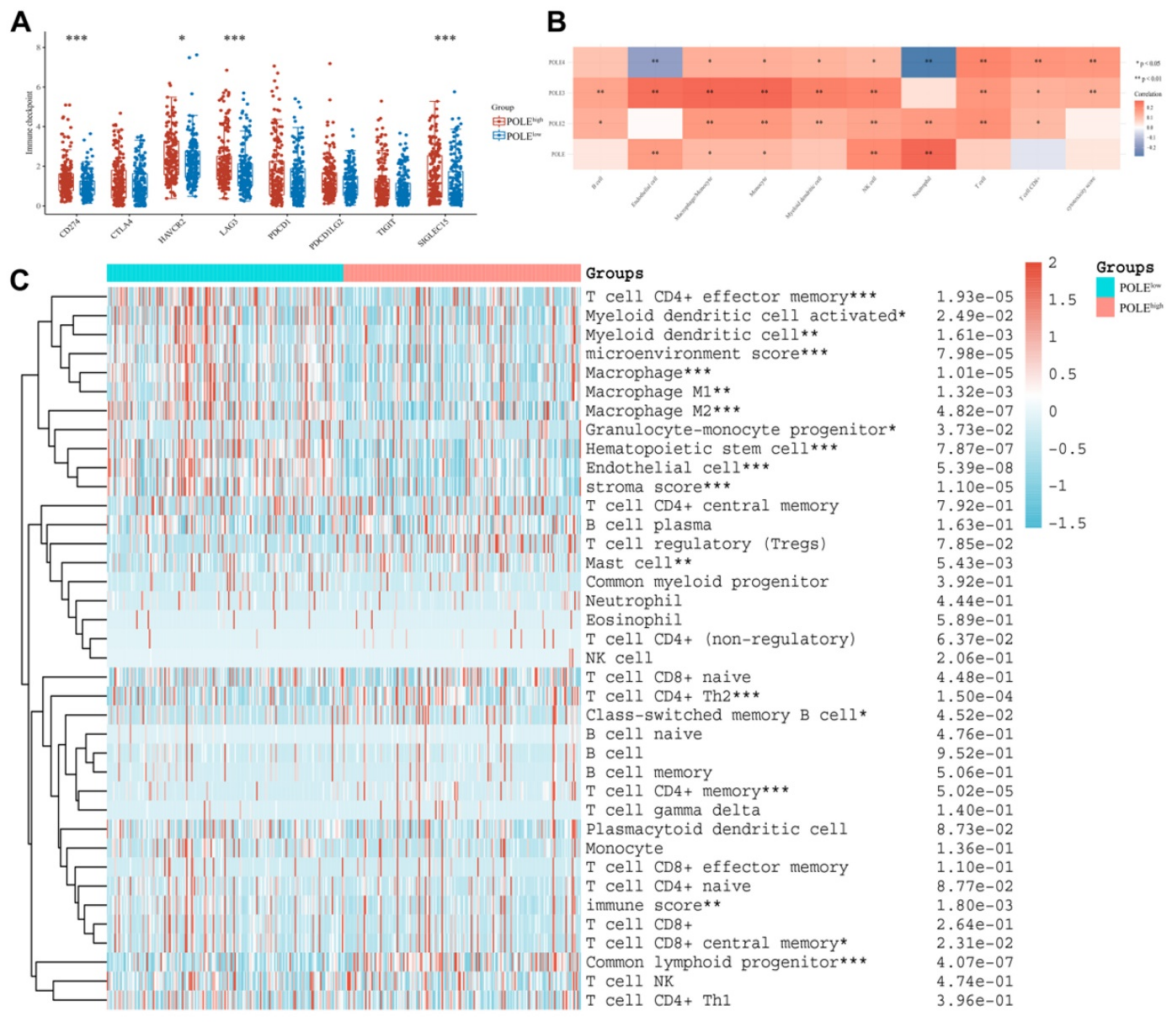
** Immunological response within HCC in accordance with POLE expression.(A)** Several checkpoints expression level under high CCL19 expression and low expression in HCC were chosen and compared. **(B)** The graph reflects the connection between different immune cells and POLE families. Red represents positive correlation while blue represents negative correlation. **(C)** We investigated the correlation of POLE expression level with different immune cells in pan-cancer and displayed it with a heat map.

Finally, we explored the possible association between POLE expression and the immune system. High POLE expression corresponded to increased immune checkpoint expression, including HAVCR2, SIGLEC15 and LAG3** (Figure 8A)**. And the relationship between the POLE family and some immune cells are found out. For example, the activity of Neutrophil cells and NK cell are closely related to POLE, and the expression of Endothelial cell, Monocyte cell and Macrophage cell are related to POLE3 expression **(Figure 8B)**. HCC patients were clarified as POLE^high^ group and POLE^low^ group. The immune cells, especially T cell CD4+ effector memory, Macrophage and Hematopoietic stem cell, were significantly less active in POLE^high^ group than those in POLE^low^ group, suggesting that the immune cells may not function well in the environment of high POLE expression, which also leads to the deterioration of the tumor **(Figure 8C)**.

## Discussion

HCC is a global cancer with high mortality and high recurrence rate. Most patients are found at an advanced stage, and traditional drug therapy and other treatments have adverse outcomes such as drug resistance 23. Immune checkpoints for immunotherapy are also being explored 13, 24. So, we focused on POLE to further study the early diagnosis and treatment of HCC. In this study, we performed clinicopathological, IHC and survival analyses to explore the potential prognostic value of POLE in hepatocellular carcinoma 25. We found the significant correlation between elevated POLE expression and higher grade, advanced stage, worse prognosis of HCC and a high risk of recurrence, showing that POLE is of great significance for early clinical diagnosis of HCC. Also, survival curves between different subgroups were compared and analyzed, and the prognosis of males, Asians, and vascular invasion-none groups were significantly correlated with POLE expression level. It is suggested that more in-depth research needs to be explored, and the prognosis of hepatocellular carcinoma or the expression of POLE may be affected by hormone levels or ethnic differences. Therefore, we have verified our hypothesis that POLE expression level can be used as a promising biomarker for prognosis of HCC.

Then, using the KEGG and GO functional annotations enrichment analyses, we found pathways involved in up-regulated genes and down-regulated genes. Besides, in the POLE^high^ group, the expression of some protein coding genes was also increased, such as SMC4, ECT2 and NLN, while in the POLE^low^ group, there were more down-regulated gene, like CYP2E1, HPR and C9. Therefore, the expression of POLE may change the expression of related genes, which may affect the effect of immunotherapy. For example, C9 gene is a component of membrane attack complex (MAC), which plays a key role in innate and adaptive immune response by forming pores in the plasma membrane of target cells 26. In addition, we explored the potential role of POLE mutation and related genes. The highest mutation rate was found in TP53, a pathway implicated in HCC carcinogenesis. And its mutation was more significant in POLE high expression group than POLE low expression group, indicating the prognostic value of TP53 expression level in HCC 27-29.

POLE is involved in DNA replication and has recently been identified as an inherited cancer susceptibility gene because its alterations are associated with colorectal cancer and other tumors 30. POLE-related syndromes show an autosomal dominant inheritance pattern. Although the prevalence of germline monoallelic POLE pathogenic variant is low, they identify distinct phenotypes with a broad tumor spectrum, in contrast to other genetic disorders such as Lynch syndrome or familial adenomatous polyposis 31. Endometrial and breast cancers, and possibly ovarian and brain tumors, are also associated with POLE alterations. POLE-mutated colorectal and endometrial cancers are associated with better prognosis and may exhibit favorable responses to immunotherapy 32. Since POLE-mutated tumors display a high tumor mutational burden, resulting in an increase in neoantigens, the identification of POLE alterations can aid in the selection of patients suitable for immunotherapy 33, 34.

Insufficient metabolic reprogramming of the immunosuppressive TME may limit the recovery of antitumor immunity, leading to malignant tumor progression. To investigate the relationship between POLE and immune microenvironment, we employed the complex machine learning algorithm, and found that POLE is closely correlated with the expression of Macrophage cells and T cell CD4+ effector memory. As a marker for early detection of hepatocellular carcinoma, POLE may also promote the activity of these immune cells and strengthen the phagocytosis of cancer cells.

However, there exist some limitations of this research. First, we did not conduct prospective cohort trial to verify the effect of POLE expression on recurrence and long-term survival in HCC patients. To compensate, we included 130 samples of HCC patients and grouped them by identifying the expression of POLE by IHC method. High POLE expression had significant correlation with worse OS (*p*=0.036, HR=1.893) and PFS (*p<0.001*, HR=3.089). Second, no experimental study was done to explore the biological function of POLE in HCC tissues or cells. However, through high-throughput dataset screening, we identified and discovered pathways in KEGG and GO databases, and performed functional enrichment analysis (Figure 6C). In addition, we studied the effect of POLE expression on the tumor immune environment, expressed through the anti-tumors' activity of immune cells. POLE is associated with increased expression of immune checkpoints and activation of immune cells, suggesting that HCC patients with high POLE expression are more suitable for immunotherapy.

## Conclusion

In conclusion, this study first revealed that altered expression of POLE in HCC significantly correlated to the malignant progression and anti-tumoral activity of immune cells. Thus, POLE could function as a predictive biomarker for the early diagnosis, aggressive progression, and immune-excluded tumor microenvironment of HCC, especially the for Asian, male, low-risk HCC patients.

### Ethical Approval and Consent to participate

All of the study designs and test procedures were performed in accordance with the Helsinki Declaration II. The Ethics approval and participation consent of this study was approved and agreed by the ethics committee of Renji Hospital.

### Funding statement

This study was supported by grants from the Project Funded by the Priority Academic Program Development of Jiangsu Higher Education Institutions.

### Data sharing statement

Data used in this article can be publically obtained, which was detailed Materials and methods.

### Author contributions

WRL, HJT and WHX carried out the molecular genetic studies, participated in the sequence alignment and drafted the manuscript. FJX, SYW and XXH carried out the immunoassays. SYW, KFL and WHX participated in the sequence alignment. WRL and HNH, HLZ participated in the design of the study and performed the statistical analyses. YX, DDS and HDT conceived the study, participated in the study design and coordination and helped to draft the manuscript. All authors read and approved the final manuscript.
